# A customized high-resolution array-comparative genomic hybridization to explore copy number variations in Parkinson’s disease

**DOI:** 10.1007/s10048-016-0494-0

**Published:** 2016-09-17

**Authors:** Valentina La Cognata, Giovanna Morello, Giulia Gentile, Velia D’Agata, Chiara Criscuolo, Francesca Cavalcanti, Sebastiano Cavallaro

**Affiliations:** 1 Institute of Neurological Sciences, National Research Council, Catania, Italy; 2 Department of Biomedical and Biotechnological Sciences, Section of Human Anatomy and Histology, University of Catania, Catania, Italy; 3 Department of Neurosciences, Reproductive and Odontostomatological Sciences, Federico II University, Naples, Italy; 4 Institute of Neurological Sciences, National Research Council, Mangone (CS), Italy

**Keywords:** aCGH, CNVs, Parkinson’s disease, Neurological disorders, Genes

## Abstract

Parkinson’s disease (PD), the second most common progressive neurodegenerative disorder, was long believed to be a non-genetic sporadic syndrome. Today, only a small percentage of PD cases with genetic inheritance patterns are known, often complicated by reduced penetrance and variable expressivity. The few well-characterized Mendelian genes, together with a number of risk factors, contribute to the major sporadic forms of the disease, thus delineating an intricate genetic profile at the basis of this debilitating and incurable condition. Along with single nucleotide changes, gene-dosage abnormalities and copy number variations (CNVs) have emerged as significant disease-causing mutations in PD. However, due to their size variability and to the quantitative nature of the assay, CNV genotyping is particularly challenging. For this reason, innovative high-throughput platforms and bioinformatics algorithms are increasingly replacing classical CNV detection methods. Here, we report the design strategy, development, validation and implementation of *NeuroArray*, a customized exon-centric high-resolution array-based comparative genomic hybridization (aCGH) tailored to detect single/multi-exon deletions and duplications in a large panel of PD-related genes. This targeted design allows for a focused evaluation of structural imbalances in clinically relevant PD genes, combining exon-level resolution with genome-wide coverage. The *NeuroArray* platform may offer new insights in elucidating inherited potential or de novo structural alterations in PD patients and investigating new candidate genes.

**Electronic supplementary material:**

The online version of this article (doi:10.1007/s10048-016-0494-0) contains supplementary material, which is available to authorized users.

## Introduction

Parkinson’s disease (PD) is a progressive debilitating movement disorder that affects approximately 1 % of the population older than 65 years of age worldwide [1]. Clinically, most patients present resting tremor, bradykinesia, stiffness of movement and postural instability. These major symptoms derive from the profound and selective loss of dopaminergic neurons in the substantia nigra pars compacta (SNc), coupled with the accumulation of eosinophilic intracytoplasmic aggregates termed Lewy bodies (LBs) [1]. Like other complex diseases, PD is believed to be a multifactorial syndrome, resulting from an elaborate interplay of numerous elements (genes, susceptibility alleles, environmental exposures and gene-environment interactions), and its molecular aetiology remains incompletely understood [2].

In recent years, the intensive efforts of the scientific community and the significant and rapid advancement of biotechnologies have fuelled several steps towards the elucidation of the genetic components of PD. Genome-wide linkage scans and exome sequencing of well-characterized PD families have been successful in discovering disease-causing mutations in dominant (*SNCA*, *LRRK2*, *VPS35* and the recent *TMEM230*), recessive (*PARK2*, *PINK1*, *DJ1*, *DNAJC6*) [2–4] and X-linked (*RAB39B*) PD genes [5, 6]. Other genes, such as *CHCHD2* and *EIF4G1*, are associated with familial PD inheritance but still require independent confirmations [7, 8]. Moreover, a set of genes related to atypical parkinsonian forms is known and includes *ATP13A2*, whose mutations cause the Kufor-Rakeb syndrome (PARK9) [9]. Despite the existence of these rare Mendelian monogenic forms, it is now clear that PD is a genetically heterogeneous and most likely complex disorder. This complexity is underlined by the notion that we are currently aware of dozens of loci, genes and risk factors that seem to contribute to PD [2, 10]. These genes are involved in numerous cellular pathways, such as the ubiquitin-proteasome system, synaptic transmission, autophagy, lysosomal autophagy, endosomal trafficking, mitochondrial metabolism, apoptosis and inflammatory mechanisms, all of which are generally implicated in neuronal cell death [11].

While the major pathogenic mutations are single nucleotide polymorphisms (SNPs) in the coding regions of PD-linked genes, the contribution of other types of DNA molecular defects (e.g. structural chromosome abnormalities such as CNVs) to the genomic architecture is less emphasized but equally significant [12, 13]. CNVs are unbalanced rearrangements larger than 50 bp and arise from genomic instability [12]. They are recognized as critical elements for the development and maintenance of the nervous system and appear to contribute to hereditable or sporadic neurological diseases, including neuropathies, epilepsy, autistic syndromes, psychiatric illnesses and neurodegenerative diseases, such as PD [14–16]. In this regard, several CNVs have been reported in PD patients, including specific pathogenic anomalies mapped in PD loci or involving candidate PD-related genes [17]. To mention the most recurrent, *SNCA* copy-number gains have been proven to play a major role in the disease severity of PARK1, while *PARK2* homozygous or compound heterozygous exon copy number changes are very common among the early-onset cases, rendering the gene-dosage assay essential in mutational screening.

Currently, the detection of CNVs and gene dosage imbalances mainly relies on traditional methodological approaches (karyotyping and PCR-based approaches such as quantitative PCR and multiple ligation probe analysis). However, these methodologies bear objective limits: they are time-consuming and labour-intensive, require multiple phase steps and severe equipment costs and, above all, do not provide a complete genomic overview of structural imbalances at sufficiently high resolution. The development of the array-based comparative genomic hybridization (aCGH) technology has dramatically improved and catalysed the detection and characterization of multiple CNVs, offering high reproducibility, high resolution and scalability for complete genome-wide mapping of imbalances [18]. The aCGH technique has been refined to the most advanced aCGH plus SNP edition, a widely used array able to simultaneously perform SNP genotyping and CNV detection. This methodology shows higher sensitivity for the detection of low-level mosaic aneuploidies and chimerism and offers the ability to detect loss of heterozygosity, but it has a limited ability to detect single-exon CNVs due to the distribution of SNPs across the genome. For this reason, several customized aCGHs suitably designed to focus on specific clinically relevant chromosomal locations have been developed and are already applied to different human diseases, including neuromuscular diseases, cancer, autism, epilepsy, multiple sclerosis, mitochondrial and metabolic disorders [19–24].

In this study, we developed a customized exon-centric aCGH (hereafter called *NeuroArray*), tailored to detect single/multi-exon deletions and duplications in a large panel of PD-related genes. We will first report the design strategy and the applied analysis methods. Then, we will show two representative PD cases tested on *NeuroArray*. Our findings show the advantages of the *NeuroArray* platform in terms of results, time and costs, as well as for the discovery of new potential genetic biomarkers underlying the pathogenic mechanisms of PD and commonly shared genetic signatures with other neurological diseases.

## Materials and methods

### Gene selection and aCGH design strategy

To build the customized *NeuroArray* aCGH platform, we aimed to obtain a high-density probe coverage in the coding region of clinically relevant genes associated with PD. Gene selection relied on our expertise in the clinic, genetics and literature data and has been extended to the entire currently known sets of genes collected in PDGene (http://www.pdgene.org/) [25]. The list of selected genes embraces disease-causing genes, known and putative risk factors and other genetic regions affected by different types of mutations. To perform a differential diagnosis, we also included genes related to other neurological conditions (see Supplementary Information and Supplementary Tables).

The array design was carried out by using the web-based Agilent SureDesign Software (Agilent Technologies, Santa Clara, CA), a web application that allows one to define regions of interest and select the “best-performing” probes from the High-Density (HD) Agilent probe library. Candidate probes were scored and filtered using bioinformatics prediction criteria for probe sensitivity, specificity and responsiveness under appropriate conditions. We also selected a limited number of probes by genomic tiling to cover regions inadequately represented in the Agilent database. All probes had similar characteristics: isothermal probes, with melting temperature (Tm) of 80 °C and probe length of approximately 60-mers, in accordance with the manufacturer’s specifications. Further details about the design method, the number of genes and exons, the median probe spacing and other characteristics of *NeuroArray* are summarized in Table 1, Supplementary Information and Supplementary Table 1.

**Table 1:** Main characteristics of the customized PD panel

Customized PD panel design	
Total genes	505
Total exonic targets	6826
Target coverage	94 %
Total target/exon size	1935 Mbp
Total probes (1–2 probes per exon)	11,161
Total unique probes from HD database	10,411
Total unique probes by genomic tiling	750
Median probe spacing	391 bp
Mean target size	323 bp
Uncovered targets	431

The table lists the total number of selected genes and exon targets, the mean exon size, the number of probes, the median probe spacing and the total coverage of the customized design for CNV detection in PD. The array design was performed through the Agilent SureDesign software (https://earray.chem.agilent.com/suredesign/). The majority of probes have been scored and filtered from the High-Density (HD) Agilent probe library. A limited number of probes have been designed with the Genomic Tiling option to cover regions inadequately represented in the Agilent database. All probes have been chosen with similar characteristics: isothermal probes, with melting temperature (Tm) of 80 °C and probe length of ~ 60-mers

### Clinical sample selection

To validate the *NeuroArray*, we selected DNA samples from individuals suffering from PD or other neurological disorders and previously subjected to gene dosage through multiplex ligation-dependent probe amplification (MLPA), quantitative real-time polymerase chain reaction (qPCR) or other commercially available whole-genome aCGH. Moreover, DNA samples of patients with PD phenotypes but an incomplete molecular diagnosis were referred for *NeuroArray* molecular cytogenetic testing. Informed consent was obtained for the use of DNA samples and for the access to medical records for research purposes.

### Microarray experiment and data analysis

Genomic DNA was extracted from peripheral blood lymphocytes using the EZ1 DNA Blood extraction kit (Qiagen, Hilden, Germany) by the BioRobot EZ1 following the manufacturer’s recommendations (Qiagen, Hilden, Germany). Highly concentrated DNA was checked for quality using the NanoDrop spectrophotometer (Thermo Scientific, Wilmington, DE). Array experiments were performed as recommended by the manufacturer (Agilent Technologies, Santa Clara, CA), and data were extracted using Feature Extraction software (Agilent Technologies, Santa Clara, CA). After the quality control check, data visualization and analysis were performed with CytoGenomics software v. 3.0.6.6. (Agilent Technologies, Santa Clara, CA) using both ADM-2 and ADM-1 algorithms. Moreover, we took into account a single-probe analysis to include putative exonic variants. Significant single exonic probe signals were clustered for pathologies according to their location on causative or susceptibility genes through a homemade script on R-platform [26]. Full details on microarray experiments and data analysis are available in the Supplementary Information.

### Validation

Ad hoc qPCR assays were performed to validate genomic imbalances detected by the *NeuroArray* as previously described [27]. Primers flanking the putative exonic imbalances were designed using the Primer-BLAST tool (http://www.ncbi.nlm.nih.gov/tools/primer-blast/). Each qPCR assay was performed in triplicate using the LightCycler 1.5 (Roche Diagnostics, Germany). The relative quantification was measured using the ∆∆Ct method, which requires a healthy control sample (diploid) as a calibrator in all amplifications [28]. As a calibrator control, we used the same DNA reference hybridized in the *NeuroArray* experiments. A control gene, checked as normal double copies on *NeuroArray*, was used as a reference for normalization. We considered a ΔΔCt value ≤0.6 as a loss, included from 0.8 to 1.2 as normal diploid, and ≥1.4 as a gain. PCR products were visualized by agarose gel electrophoresis.

## Results

### aCGH design on a targeted PD gene panel

**Fig. 1:**
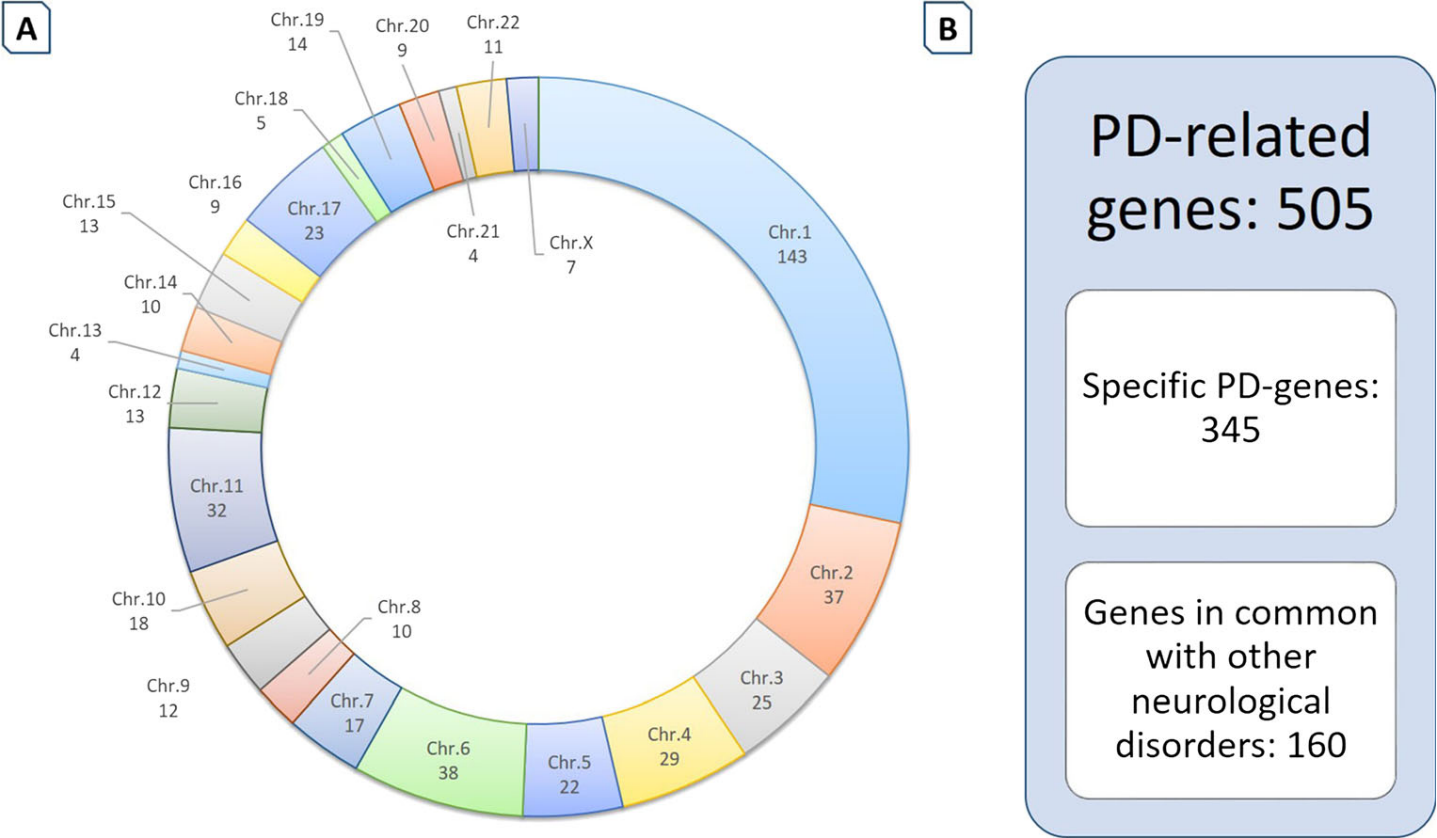
Distribution of selected PD genes on the human genome and overlap with other neurological diseases. **a** Graphical representation showing the number of clinically relevant genes for chromosomes included in the customized PD panel. The total number of selected genes is 505, mostly enclosed in chromosome 1. Chromosome Y does not include PD-related genes. **b** The PD panel globally targets 505 PD-related genes. Of these, 345 are specific for PD, while 160 are in common with other neurological diseases. These latter ones can be useful to study the potential overlapping genetic signatures among different neurological conditions and to better define the genotype/phenotype correlations

To perform a comprehensive analysis of CNVs in PD-related genes, we developed a focused customized oligonucleotide aCGH design targeting 505 genes and 6826 exonic regions linked to PD. Overall, 11,161 probes with a median probe spacing of 391 bp were enriched in the coding regions of these genes (Table 1). The majority of targeted genes map on chromosome 1, while lower numbers are distributed among the other chromosomes (Fig. 1).

**Fig. 2:**
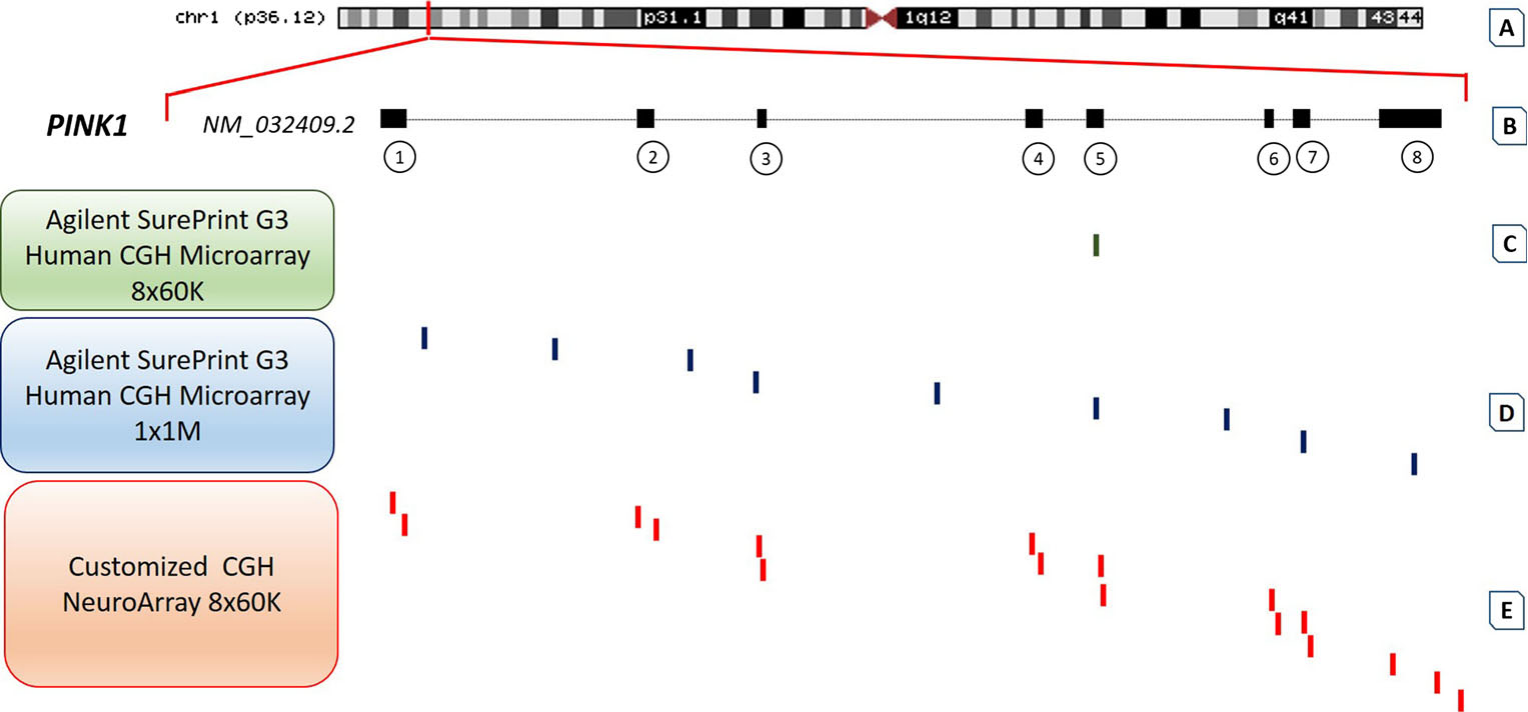
Oligonucleotide probe distribution on *PINK1* in different commercially available whole-genome aCGH platforms and *NeuroArray*. **a** The human *PINK1* gene is located on chromosome 1 (cytoband p36.12), spanning approximately 18 kb of genomic DNA. **b** This gene produces an mRNA transcript encompassing eight exonic regions (NCBI accession number NM_032409.2). Exons are represented in the figure by *black boxes* and are numbered consecutively. The *gray line* represents intronic regions. **c** Distribution of oligonucleotide probes (*green bars*) in the commercially available whole-genome Agilent SurePrint G3 Human CGH Microarray 8 × 60K. As evidenced in the figure, this platform has just one probe overlapping *PINK1* exon 5, proving low-resolution coverage. **d** Distribution of oligonucleotide probes (*blue bars*) in the whole-genome Agilent SurePrint G3 Human CGH Microarray 1 × 1M slide format. The highest-resolution 1 × 1M array CGH reveals the *PINK1* genetic region with a greater number of oligonucleotide probes; however, it is five times more expensive per sample than the Agilent 8 × 60K slide format and leaves uncovered some exonic traits (for example, exon 1 or 2). **e** Distribution of oligonucleotide probes (*red bars*) in the entire exonic regions of the *PINK1* gene in the customized *NeuroArray* design. The *NeuroArray* design allows high-density probe enrichment in the entire exonic regions of *PINK1*, enabling a focused evaluation of structural imbalances at a single-exon resolution with costs comparable to an 8 × 60K slide format. (Colour figure online)

The tightly restricted criteria used for the array customization have allowed a higher exonic probe enrichment on selected gene panels, overcoming the resolution of commercially available genome-wide CGH array platforms. Overall, 94 % of the total exon targets are covered by at least one probe in the *NeuroArray* design (Table 1), while other commercially available aCGH platforms provide a lower probe coverage of the same selected exonic regions. For example, the Agilent SurePrint G3 Human CGH Microarray 8 × 60K slide format covers our selected regions by 8.2 %, while the highest-resolution 1 × 1M array provides 25 % of our target coverage. A representative illustration is reported in Fig. 2 and focuses on *PINK1* (RefSeq acc. no. *NM_032409.2*).

To perform an accurate differential analysis between PD patients and other neurological phenotypes, we also included genes related to other neurological disorders (Supplementary Information and Supplementary Tables **).** Specifically, 160 of the 505 PD-related genes were linked to other neurological conditions (Fig. 1).

### CNVs of PD-related genes detected through the *NeuroArray* platform

*NeuroArray* was able to confirm copy number changes previously characterized by other methodological strategies and revealed new interesting genomic imbalances. In the following sections, we will show two representative examples of *NeuroArray* tests obtained by using genomic DNA samples of PD patients. Further CNVs were observed in other neurological disease-related panels and were validated by qPCR (data not shown).

#### Application of an integrated ADM-1 and ADM-2 algorithm-based data analysis to improve CNV calling

The DNA sample of patient no. 1 was referred to our laboratory for molecular testing of *PARK2*, *PINK1* and *DJ1*, to confirm the clinical diagnosis of familial recessive early-onset PD. Mutation analysis showed a heterozygous C1305T single nucleotide substitution in the coding region of *PARK2*. *NeuroArray* (with the ADM-1 algorithm) revealed 10 different CNVs, overall composed of 6 gains and 4 losses (Table 2). Four of them included genes previously linked to PD [29–32], while the others overlapped with genes related to other neurological conditions [33–42] (Table 2).

**Table 2:** Representative view of genomic CNVs detected by *NeuroArray* in a PD patient

CNV type	Chr	Start–stop (bp)	Size (kb)	Cytoband	No. of probes	Annotations	Disease-linked genes	Ref.	Previously described in DGV?	ClinGen nomenclature (described pathogenic variants)
Gain	1	6,579,851–8,021,801	1442	p36.31–p36.23	3	PLEKHG5, NOL9, TAS1R1, ZBTB48, THAP3, DNAJC11, KLHL21, PHF13, CAMTA1, VAMP3, PER3, UTS2, TNFRSF9, **PARK7**	PLEKHG5 (neuropathies and ALS), PARK7 (**PD** and ALS)	[2, 39–41]	Overlaps nsv1004598	Almost completely contained within nssv1602095, nssv578510, nssv578509
Gain	1	54,704,828–54,747,170	42	p32.3	12	**SSBP3**	**PD**	[29]	No	Completely contained within nssv578523 and nssv578522
Gain	2	179,541,928–179,542,606	1	q31.2	3	TTN	Limb-girdle muscular dystrophy	[38]	No	Not reported
Loss	3	142,216,000–142,222,244	6	q23	6	ATR	Epilepsy	[42]	Overlaps nsv528954	Completely contained within nssv583804, nssv577921, nssv577927, nssv583018, nssv1602712, nssv3395109
Loss	3	178,922,270–178,927,435	5	q26.32	3	PIK3CA	Epilepsy	[37]	No	Not reported
Gain	5	70,307,077–70,308,602	2	q13.2	4	NAIP	ALS	[36]	Yes (completely contained in several genomic structural variants)	Completely contained within nssv1602328
Gain	9	131,012,433–131,314,975	303	q34.11	6	DNM1, GOLGA2, C9orf119, TRUB2, COQ4, SLC27A4, URM1, CERCAM, ODF2, GLE1, SPTAN1	DNM1 (epilepsy), SPTAN1 (epilepsy)	[34, 35]	Overlaps nsv1051081	Completely contained within nssv579112, nssv579118, nssv579123, nssv579147, nssv579149, nssv576650, nssv584344, nssv584434, nssv579136, nssv579139, nssv579121, nssv579124, nssv579127, nssv579138, nssv579140, nssv1602335, nssv1494937, nssv1415412, nssv1603388, nssv3397050, nssv3397066, nssv3397108
Loss	9	139,903,473–139,904,058	1	q34.3	4	ABCA2	AD	[33]	Yes (completely contained in several genomic structural variants)	Completely contained within nssv579112, nssv579118, nssv579123, nssv579147, nssv579149, nssv576610, nssv576650, nssv584344, nssv584430, nssv584434, nssv579136, nssv579139, nssv1603388, nssv1604614, nssv3397050, nssv3397066, nssv3397108, nssv579121, nssv579124, nssv579127, nssv579138, nssv579145, nssv707149, nssv582161, nssv1602986, nssv1494937, nssv1415412, nssv1602335, nssv1494929
Gain	12	56,615,423–56,615,694	0.27	q13.3	3	**RNF41**	**PD**	[30]	Yes (completely contained within nsv1051961)	Completely contained with nssv1603959
Loss	17	44,701,610–44,771,900	70	q21.31	14	**NSF**, NSFP1	**PD**	[45]	Yes (completely contained in several genomic structural variants)	Not reported

The *NeuroArray* aCGH (ADM-1 analysis method) revealed 10 different CNVs overall composed of six gains and four losses. Four of them overlapped with genes belonging to the PD panel (in bold type), while the others mainly included ALS or epilepsy-related genes. Overlapping structural variants previously described and deposited in DGV (http://dgv.tcag.ca/) or ClinGen (https://www.clinicalgenome.org/) databases are also indicated. Start and stop coordinates refer to UCSC Genome Browser Human Feb. 2009 Assembly (GRCh37/hg19)

**Fig. 3:**
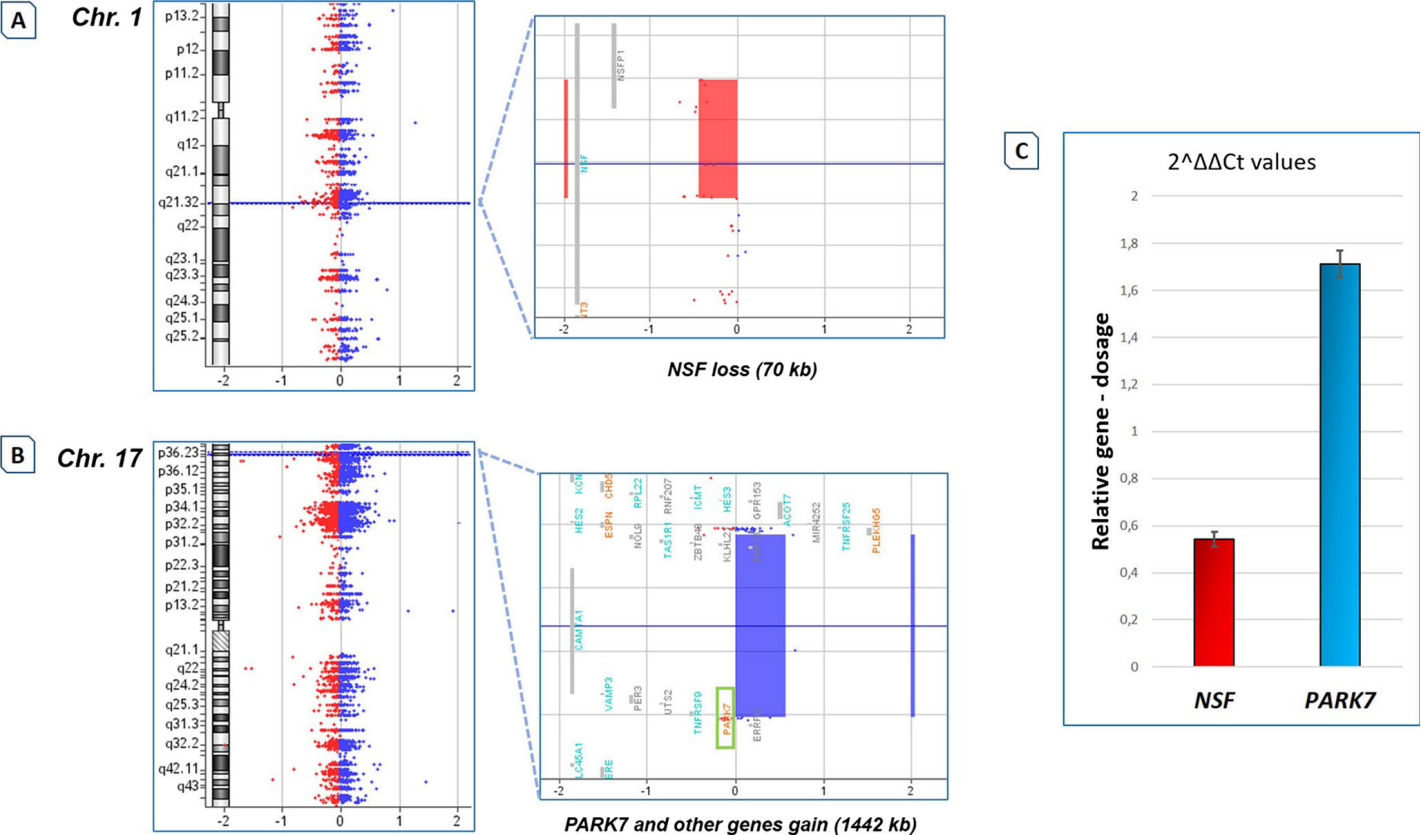
A representative example of CNV detection involving PD-related genes in a patient with early-onset PD. The *NeuroArray* platform detected several CNVs in a female patient with early-onset PD and a mild phenotype (the reader is also referred to Table 2). **a** Visualization of the *NSF* deletion detected by *NeuroArray* as shown by CytoGenomics software. The *left panel* shows the entire chromosome 1, while the *right panel* is a *zoom-in* of the deleted region (indicated by the *red area*). *Red* and *blue dots* represent the log2 ratios for the relative hybridization intensities of each spotted probe. **b** Visualization of the *PARK7* amplification detected by *NeuroArray* as shown by CytoGenomics software. The *left panel* shows the entire chromosome 17, while the *right panel* is a *zoom-in* of the amplified region (indicated by the *blue area*). For *red* and *blue dots*, see **a**. *Dots* with an average log2 ratio of approximately +0.58 indicate a heterozygous amplification. **c** Validation of both CNVs of *NSF* and *PARK7* by qPCR. Relative gene dosage levels of *NSF* and *PARK7* genes are based on delta Ct calculation. Ct values of both genes were normalized to the Ct value of a normal diploid gene. The relative level of each gene of interest is presented as the mean of 2^−ΔΔCt^, as described in the “Methods” section. *Error bars* indicate standard deviations from the mean. (Colour figure online)

The most interesting findings regarded two principal dosage anomalies: (i) the gain of a 1442-kb region on chromosome 1, which encompasses *PARK7*, and (ii) the loss of the *NSF* (*N*-ethylmaleimide-sensitive factor) gene on chromosome 17 (Fig. 3a, b). Mutations in *PARK7* comprehensively account for ∼1 % of the early-onset familial cases [1], and its copy number changes have been previously observed in PD patients [43, 44]. *NSF* is involved in vesicular trafficking, membrane fusion and synaptic neurotransmission, and its genetic alterations (both SNPs and deletion) have been previously reported in PD patients [31, 45]. Validations of these genomic rearrangements were performed with qPCR assays, suitably designed to target *PARK7* exon 1 and *NSF* exon 11. Both assays confirmed the CNVs with 100 % concordance and confirmed the heterozygous gain/loss (Fig. 3c). Primer sequences and PCR conditions are available upon request.

It should be highlighted that the default analysis with the ADM-2 algorithm revealed the loss of only the *NSF* gene. If this method were the only one applied, other relevant real CNVs (like the *PARK7* gain, later confirmed by qPCR) would have been lost. On the other hand, the analysis with ADM-2 allowed for the filtering of possible false-positive CNVs within the ADM-1 analysis. It appears important, therefore, to integrate data from both CNV calling algorithms in order to provide a more accurate data analysis and, consequently, ensure a more effective quality assessment and experimental validation.

#### Detection of single-exon copy number changes by *NeuroArray*

Although some authors have outlined the evidence that a significant proportion of single probe intervals represents real events [46], in aCGH studies, it is often recommended to report only intervals detected by three or more consecutive probes. Due to this approach, deletions or duplications below certain size cut-offs are usually ignored in the aCGH reports and not reported. However, these genomic alterations (detected by less than three probes) have been demonstrated to be definitively crucial for particular clinical diagnoses [47]. Along this line, we applied a single probe analysis to reveal short genomic imbalances in the exonic regions of strongly linked causative genes. The utility of this approach on *NeuroArray* data analysis is shown in the following case.

**Fig. 4:**
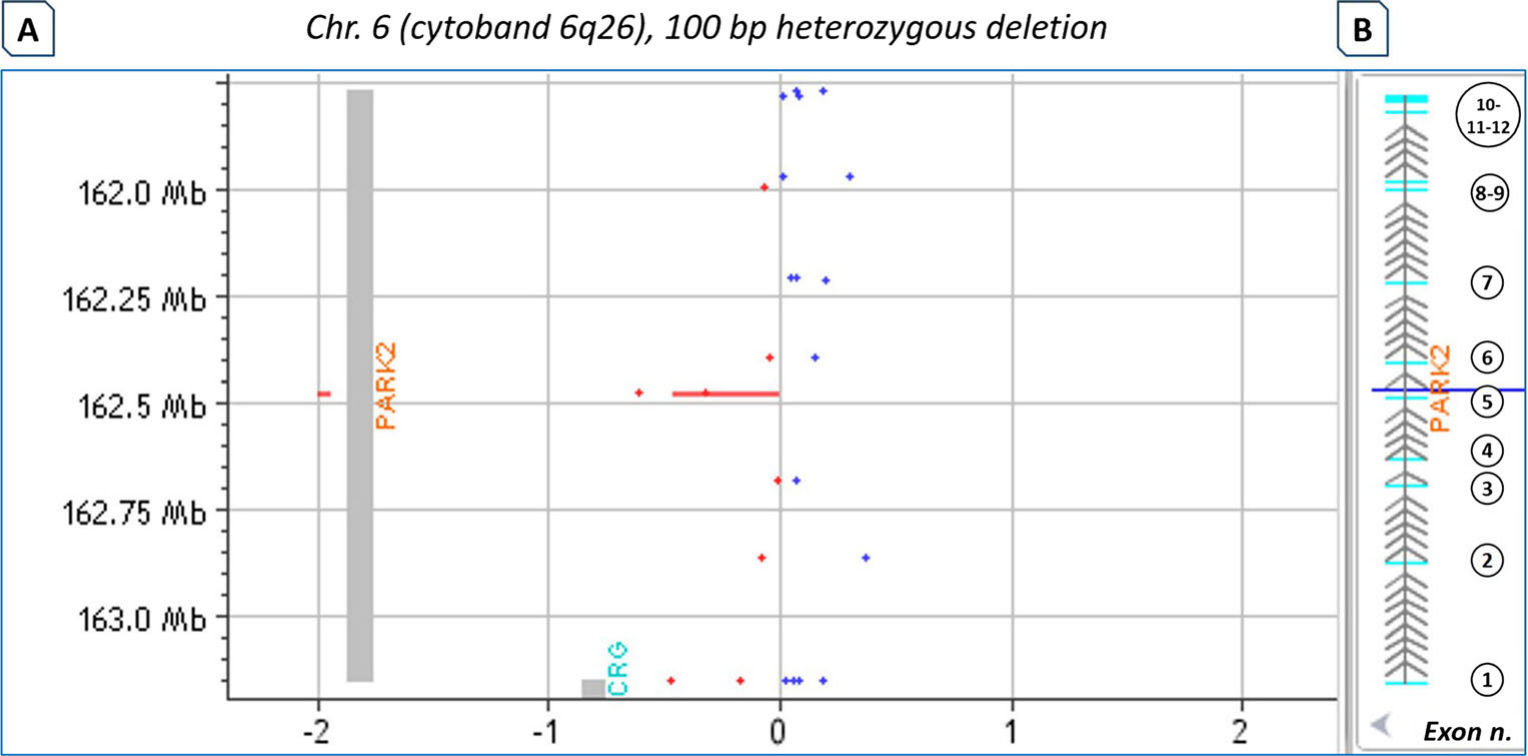
Detection of intragenic *PARK2* deletion (exon 5) in a patient with autosomal juvenile Parkinson’s disease. Heterozygous deletion of exon 5 of the *PARK2* gene detected by *NeuroArray* in a patient with juvenile Parkinson’s disease (PD) and previously revealed by an MLPA assay. **a** *NeuroArray* aCGH data visualization and analysis as shown by CytoGenomics software. The *red area* represents the deleted region. The *top* of the *panel* shows the size of the deletion and the chromosomal locus. *Red* and *blue dots* represent the log2 ratios for the relative hybridization intensities of each spotted probe. The *dots* with an average log2 ratio around −1 indicate a heterozygous deletion. **b** The panel displays the *PARK2* gene as annotated in the UCSC Genome Browser Feb. 2009 GRCh37/hg19 (https://genome-euro.ucsc.edu). *Blue boxes* represent exons and are numbered consecutively, whereas *grey arrows* are the intronic regions. (Colour figure online)

Patient no. 2 was a sporadic PD patient, carrying a heterozygous deletion of two adjacent exons (4 and 5) of the *PARK2* gene. This deletion was previously revealed by an MLPA assay (SALSA MLPA Kit P051/P052 Parkinson; MRC-Holland). The *NeuroArray* test was able to detect and confirm the deletion of exon 5 through two consecutive probes (Fig. 4) but was not able to detect the exon 4 deletion because during the phase of array design, this exon skipped the optimum parameters for probe coverage. The total concordance with the MLPA test was 91 %. Despite this limit, the one-probe analysis was essential to detect the exon 5 *PARK2* deletion, which otherwise would not have been properly outlined using the analysis of three consecutive probes. However, this approach may result in a great number of false positives. Therefore, it is advisable to use it as a validation strategy for previously known exonic imbalances, i.e. next generation sequencing (NGS)-targeted panels, or to investigate copy number changes in a small set of strongly causative genes.

## Discussion

In recent years, several studies have highlighted the key role of CNVs in the development of hereditable or sporadic neurological diseases, including PD [14–16]. Many gene-dosage anomalies have been previously mapped in PD patients, including familiar genes (*SNCA*, *PARK2*, *PINK1*, *PARK7*, *ATP13A2*) [48, 49], as well as several rare CNVs in candidate regions [45]. The aCGH biotechnology currently represents a useful tool for the detection of unbalanced chromosomal changes across the human genome, and its applications to screen common benign and rare pathogenetic CNVs are extensively growing [19–23]. The classical methodologic approaches are a gold-standard test when applied to monogenic disorders, but when applied to multigenic complex pathologies (such as PD), they require higher equipment costs, time, steps and personnel [50]. Conversely, targeted aCGH is rapid, relatively inexpensive, highly sensitive and an accurate method to simultaneously detect single- and multi-exon CNVs in numerous genes on a unique common platform. For this reason, several whole-genome and exon-targeted aCGH platforms have already been implemented in human diseases [19–24], and their utility has been demonstrated in patients with various clinical complex phenotypes [51–53].

In this study, we have designed and validated a targeted exon-centric aCGH platform (*NeuroArray*) as a molecular testing tool to simultaneously screen CNV imbalances in a large set of clinically relevant genes for PD and other complex neurological diseases. This customized design offers some considerable advantages: it allows an exon-focused evaluation of structural imbalances in clinically relevant regions at a higher resolution than whole-genome commercially available platforms and lowers the costs of an “exon by exon” analysis through PCR-based approaches, simultaneously providing an extensive window of further potentially involved genetic alterations.

In addition to the customized design, we also applied several approaches for data analysis. The first interesting result was the need to integrate data from both the ADM-1 and ADM-2 algorithms for CNV calling aberrations in order to reduce the number of false positives and to bring out relevant CNVs that otherwise would have been lost. We have also employed a one-probe analysis to reveal small imbalances at the single-exon level. Although this approach has the potential to detect crucial genetic variations ignored by multi-probe analysis, it largely increases the quantity of false-positive probe signals. Therefore, the single-probe analysis would be a useful validation strategy for NGS experiments or to investigate exon copy number changes in a smaller set of causative genes (as we performed with the script in the R-platform).

The use of dedicated high-throughput genotyping platforms like our *NeuroArray* could offer new opportunities for the PD genomic research field, mainly for familiar PD cases with an incomplete molecular diagnosis or sporadic cases without any detected genetic anomalies. The large-scale screening of genes that are involved in nervous system dysfunctions could allow for differential diagnosis with other common neurological disorders, refine the genotype-phenotype correlations and explore the potential genetic overlapping signatures among different neurological conditions [54]. Specifically, the PD panel shares a good number of genes with other neurological diseases (Fig. 1). Given the existence of PD patients with combined clinical and pathological features [55–57], this strategy could be useful to investigate common genetic anomalies underlying very complex phenotypes.

Similarly to other aCGH-based technology, *NeuroArray* has some limitations, such as the inability to detect mosaicism poorly represented, balanced structural chromosomal abnormalities, nucleotide repeat expansions (e.g. in *C9orf72* or *ATXN2* genes) and mutations included in regions not covered by probes. To overcome some of these limits and reduce the number of false-positive signals, we are developing a second version of the *NeuroArray* design with the aim of improving probe coverage in non-targeted genomic regions, including (where necessary) the intronic flanking regions and the alternatively spliced cassette exons of relevant PD genes [58–60].

## Conclusions

Our *NeuroArray* platform represents a powerful and reliable tool for the analysis of genomic imbalances associated with PD and other neurological diseases. Compared to PCR-based approaches applied to multigene analysis or to whole-genome commercially available CGH arrays, it provides a focused higher resolution at a lower cost, enabling a more detailed analysis of clinically relevant exonic regions and offering a better cost/benefit ratio. In future years, the use of this platform may offer new insights into the investigation of new genetic molecular anomalies contributing to PD, as well as a more precise definition of genotype-phenotype relationships. It may also offer novel clues in the elucidation of potential genetic overlapping among different neurological conditions.

## Electronic supplementary material

Supplementary Table 1 (DOCX 13 kb)

Supplementary Table 2 (XLSX 57 kb)

ESM 1 (DOCX 15 kb)

## Abbreviations

aCGH: Comparative genomic hybridization array
AD: Alzheimer’s disease
ALS: Amyotrophic lateral sclerosis
BMD: Becker muscular dystrophy
CNV: Copy number variation
DMD: Duchenne muscular dystrophy
HSP: Hereditary spastic paraplegia
LGMD: Limb-girdle muscular dystrophy
MLPA: Multiplex ligation-dependent probe amplification
NF: Neurofibromatosis
NGS: Next generation sequencing
PD: Parkinson’s disease
PN: Peripheral neuropathy
qPCR: Quantitative polymerase chain reaction
RTT: Rett syndrome
SCA: Spinocerebellar ataxia
TSC: Tuberous sclerosis
