# Supplementary material for: A customized high-resolution array-comparative genomic hybridization to explore copy number variations in Parkinson’s disease
Source: Neurogenetics. 2016 Sep 17;17(4):233–44. doi: 10.1007/s10048-016-0494-0 (PMC5566182; doi:10.1007/s10048-016-0494-0)
Supplement: Supplementary file 1 — (DOCX 13 kb) [file 10048_2016_494_MOESM2_ESM.docx]

**Supplementary Table 1**

**Table 1. Main characteristics of the global aCGH *NeuroArray* design**

| *NeuroArray* design | |
| --- | --- |
| Total genes | 1632 |
| Total exonic targets | 24929 |
| Target coverage | 94 % |
| Total target/exon size | 6,5 Mb |
| Total probes (1-2 probes per exon) | 40973 |
| Total unique probes from HD Database | 38636 |
| Total unique probes by genomic tiling | 2337 |
| Median probe spacing | 355 bp |
| Mean target size | 318,3 bp |
| Uncovered targets | 1520 |
| Globally disease- targeted gene panels | ALS, Epilepsies, RTT, LGMD, DMD/BMD, HSP, SCA, NF, TSC, PN, Stroke |

The table lists the total number of selected genes and exon targets, the mean exon size, the number of probes, the median probe spacing and the total coverage of the customized design. The array design was performed through the Agilent SureDesign software (https://earray.chem.agilent.com/suredesign/). The majority of probes have been scored and filtered from the High-Density (HD) Agilent probe library. A limited number of probes has been designed with the Genomic Tiling option to cover regions inadequately represented in the Agilent database. All probes have been chosen with similar characteristics: isothermal probes, with melting temperature (Tm) of 80° C and probe length of ̴ 60-mers.
