# Supplementary material for: A customized high-resolution array-comparative genomic hybridization to explore copy number variations in Parkinson’s disease
Source: Neurogenetics. 2016 Sep 17;17(4):233–44. doi: 10.1007/s10048-016-0494-0 (PMC5566182; doi:10.1007/s10048-016-0494-0)
Supplement: Supplementary file 3 — (DOCX 15 kb) [file 10048_2016_494_MOESM1_ESM.docx]

**Supplementary information on Material and Methods**

**Gene selection and aCGH design strategy.** 505 PD-related genes were selected from PDgene (<http://www.pdgene.org/>), a comprehensive, unbiased and regularly updated database of genetic association studies performed in PD (Release February 2013). In order to perform a differential diagnosis with other neurological disorders, we also included genes related to other neurological conditions, such as Amyotrophic Lateral Sclerosis (ALS), Epilepsies, Rett Syndrome (RTT), Autosomal dominant and recessive Limb-Girdle Muscular Dystrophy (LGMD), Muscular Duchenne (DMD)/Becker Dystrophy (BMD), Hereditary Spastic Paraplegia (HSP), Spinocerebellar Ataxia (SCA), Neurofibromatosis (NF), Tuberous sclerosis (TSC), Peripheral Neuropathy (PN) and Stroke. The genes related to these disorders were selected from literature or screened from other databases available online (ALZgene - <http://www.alzgene.org/> and ALSgene - http://www.alsgene.org/).

The array design was carried out by using the web-based Agilent SureDesign Software (Advanced Design Wizard option), version 1.2.1.15 (Agilent Technologies, Santa Clara, CA). This web application allows to define regions of interest and select the “best-performing” probes from the High-Density (HD) Agilent probe library. Chromosomal coordinates of all RefSeq genes were extrapolated from open-source databases, Biomart (http://www.biomart.org/) and UCSC Genome Browser according to Human Feb. 2009 Assembly (GRCh37/hg19) (http://genome.ucsc.edu). Exon coordinates were selected and formatted using a homemade R script [[1](#_ENREF_1)] and then uploaded on SureDesign. Candidate probes were scored and filtered using bioinformatics prediction criteria for probe sensitivity, specificity, and responsiveness under appropriate conditions. We also selected a limited number of probes with the SureDesign Genomic Tiling option to cover regions inadequately represented in the Agilent database. All probes had similar characteristics: isothermal probes, with melting temperature (Tm) of 80° C and probe length of about 60-mers, in accordance to the manufacturer's specifications. Biological probes were randomly distributed in an 8x60K array format that allows to process simultaneously eight samples in a single experiment. The routinely used Agilent Human CGH Normalization Probe Group (1262 features) and the Agilent Human CGH Replicate Probe Groups (5000 features) were also included in the array design. Microarray slides were produced using Agilent’s Sure-Print Inkjet technology (Agilent Technologies, Santa Clara, CA).

**Microarray experiment.** Array experiments were performed as recommended by the manufacturer (Agilent Technologies, Santa Clara, CA). DNA test and a reference of the same sex (Euro Reference, Agilent Technologies, Santa Clara, CA), both at the concentration of 500 ng, were double digested with RsaI and AluI for 2 hours at 37°C. After heat inactivation of the enzymes at 65°C for 20 min, each digested sample was labeled by random priming by using the genomic DNA Enzymatic Labelling Kit (Agilent Technologies, Santa Clara, CA) for 2 hours using Cy5-dUTP for patient DNAs and Cy3-dUTP for reference DNAs. Labeled products were column purified by using the SureTag DNA Labeling Kit Purification Columns (Agilent Technologies, Santa Clara, CA). After probe denaturation and pre-annealing with Cot-1 DNA, hybridization was performed at 65°C with rotation for 24 hr. After two washing steps, the array was scanned by SureScan scanner (Agilent Technologies, Santa Clara, CA) at 3 microns. Array data were extracted from scanned images using Feature Extraction software (Agilent Technologies, Santa Clara, CA) and underwent a quality control step in order to check signal intensities and background noise. In particular, the following evaluation metrics were used to pass our quality control test: Derivative Log Ratio Spread ≤ 0.30, signal intensity ≥200, background noise of both channels ≤ 25, and signal to noise ≥30.

**Data analysis.** A first data analysis was carried out by using the Aberration Detection Method 2 (ADM-2) algorithm with default normalization parameters. This statistical algorithm permits to identify all aberrant intervals in a given sample with consistently high or low log ratios based on a statistical score. The statistical score is calculated on the average log ratios of the probes and the number of probes per region. To make a positive call, our threshold settings for the default analysis method were 6.0 for sensitivity, 0.25 for minimum absolute average log ratio and 3 as the minimum number of probes in the region. The ADM-2 algorithm is the most stringent since incorporates quality information about each probe measurement.

A second data analysis was performed with the Aberration Detection Method 1 (ADM-1) algorithm with the threshold settings described above. ADM-1 algorithm uses the same iterative procedure as ADM-2 to find all genomic intervals with the score above a user specified threshold, but does not take into account the Quality-Weighted Interval Score, and therefore results less stringent.
